# Spencer's Psychology
*"The Principles of Psychology:" by Herbert Spencer. Longmans. 1855.


**Published:** 1857-07-01

**Authors:** 


### Art VIII.—SPENCER'S PSYCHOLOGY.*

The work to which the above heading refers, is divided into four parts, and the peculiarity—we may say originality—of its aim, appears from the first paragraph of the preface, in which the author states that each part consists of a "different view of the same great aggregate of phenomena," and is independent and complete in itself, though related intimately to all the rest; and the reader is left to his own course as to which part he may best commence with, after he is acquainted with the main drift of each part separately. The parts are entitled—"General Analysis," "Special Analysis," "General Synthesis," and "Special Synthesis."

The "General Analysis" is an inquiry into the basis of our intelligence, with the view of ascertaining, in the language of the author, "the fundamental peculiarity of all modes of consciousness constituting knowledge proper—knowledge of the highest validity." The 'Special Analysis' has for its aim to resolve each

* "The Principles of Psychology:" by Herbert Spencer. Longmans. 1855.

species of cognition into its simplest and ultimate components or constituents. Both these analytical parts of the work relate to human intelligence, merely and subjectively. In the synthetical part, Mr. Spencer proposes an object which sounds more hazardous—to "deal with the phenomena of intelligence, not human only, but under every form." We much regret, both for our sake and the author's, that the state of his health prevented his writing a "Summary and Conclusion," in which he proposed "to bring the several lines of argument to a focus." This would have much abridged the labour of forming an opinion of a book on metaphysics, of between 600 and 700 pages.

The volume opens with an inquiry respecting a "datum" for the validity of human knowledge. We receive certain axioms and postulates on the direct warrant of consciousness that they are beyond dispute. So of all the truths we call objective, whether intuitively perceived, or by strict logical deduction. But while consciousness vouches for such truths, what (asks our author) shall vouch for the validity of the dicta of consciousness? He asks for a "method of verifying our empirical cognitions," as necessary to sure results. If psychology is to become more than a mere aggregate of opinions, we must have a datum for testing the conclusions of consciousness. Logic itself rests on the validity of such criterion, and rational psychology must start from this point.

Our author maintains that the confusion and diversity which prevail on all fundamental questions and principles, are ample ground for thinking that there is some such hitherto unestablished datum. The possibility of defending "Idealism, philosophical Scepticism, Fichteism, Hegelianism," and the like theories, indicates either an error at the basis of such theories, or that reason itself leads to unreasonable conclusions. The protest of the intellect against these incongruous theories, even in those who framed them, is strong proof against them. In support of these remarks of Mr. Spencer, we may refer to the confession of Fichte, who developed in the most rigorous form the purest and most consequent theory of idealism which is to be found in the history of philosophy; and so confident was he of the correctness of his conclusions, that he on one occasion deprecated any future deviation, on his part, from the doctrines he considered himself to have established, by denouncing on himself "eternal condemnation" if he ever did so. Yet, he afterwards acknowledged that even logical proof can only be satisfactory in so far as it is based on natural belief (*Glaube*),* and that he could not believe his own idealism. Of course all such absurd theories as that which Fichte held, during his first period, and which he was always very loth to part

* *Vid.* "Bestimmung des Menschen, 130—148.

with, notwithstanding the above confession, must be founded on assumptions which are altogether untenable; and we have always thought that such an assumption was not far to seek, when we consider that it was necessary to Fichte's system to take for granted that the mind had an unconscious power of framing to itself the universe of ideas, which mankind erroneously suppose to be realities. Our author holds that such theories indicate a "non-recognition of some primordial element in our knowledge, the identification of which is all-essential." This, he argues, is shown by the failure of the efforts to overthrow these systems, for want of a fulcrum in some primary truth. Reid's argument against philosophical scepticism he regards as a protest only, not a disproof, as merely assuming what Hume rejects.

We have long thought that some of Sir W. Hamilton's remarks on "consciousness," in his valuable edition of Reid's works, are not very happy. He holds that the only possible answer to the inquiry, "How do the fundamental facts, feelings, beliefs [of consciousness], certify us of their own veracity, is that as elements of our mental constitution, as the essential conditions of our knowledge, they *must* be accepted by us as true." Now this appears to be a sort of metaphysical *hysteron-proteron;* for how are we to know that any feelings, beliefs, etc., *are* fundamental, *are* essential conditions of our knowledge, but *because* they "must be accepted?" How, for example, can we know that the belief that *every change must have a cause* is fundamental and essential, but just because we "*must*" accept the proposition—we cannot help it? If we had space, we might adduce passages from Sir William's speculations on this subject, which tend to the notion that we argue ourselves into the admission of the veracity of consciousness; but surely such argument already supposes consciousness as a primary fact. Again, the same acute Scottish metaphysician says, that "unless the melancholy fact [of the mendacity of consciousness] be proved, our faculty of knowledge is not to be supposed an instrument of illusion;" but surely such "proof" would contradict itself, by "proving" that we *can know!* Mr. Spencer has himself criticised a statement of Sir William, similar to the one we have just quoted. "Consciousness is to be presumed trustworthy until proved mendacious. The mendacity of consciousness is proved, if its data, immediately in themselves, or mediately in their necessary consequences, be shown to stand in actual contradiction." Our author acutely remarks, that this mode of reasoning is destitute of force; for "the steps by which consciousness is to be proved mendacious are themselves states of consciousness, and must be assumed as trustworthy in the very act of proving that consciousness is not so." It might have been here added, that Sir William himself has remarked, that as "doubt itself is only a manifestation of consciousness, we cannot doubt our consciousness." To us it appears evident that consciousness is final, neither admitting nor requiring any criterion. Consciousness must be true—that is, must be a fact, in any particular case of it. Its credibility must be assumed. We quite agree with the author in his views of the inconceivableness of any *test* of consciousness. The effort to apply one is, as he justly remarks, like the "mechanical absurdity of trying to lift the chair one sits on."

Those of our readers who are familiar with the writings of the late lamented Edinburgh Professor—one of the acutest, and certainly the most learned of the metaphysicians of his time—will remember the crucial argument on which he rests the defence of the philosophy of "common sense" against the Pyrrhonic and Humian scepticism, the idealism of Berkeley, and that of the Germans developed and exaggerated from the speculations of Kant. It has been a query with ourselves, believers as we are in realism, whether Sir William gains anything by his extension of the meaning of the term "conscious," when he says—"In the act of sensible perception, I am conscious of two things—of myself as the perceiving *subject*, and of an external reality, in relation to my sense, as the *object* perceived. Each of these is apprehended equally and at once in the same indivisible energy, in the same indivisible moment of intuition." At all events the sceptic might say, "I know what you mean when you say you are conscious of a sensation, conscious of a modification of your *ego*; but how can you be *conscious* of anything else than such a modification—how can you be said to be conscious of a thing which is not in the *ego*, but is external to it? You are conscious of its effect upon you—conscious of your belief in its presence and reality—conscious of the modification of the *ego* which you attribute to its agency on your organs of sense, but how conscious of what is external to your consciousness, and even to your nervous system and sensorial organs?"

The above remark relates merely to a question of strictness in the philosophical use of terms; our author's reclamation is on other grounds. He thinks that in the act of perception, "our consciousness of subject and object is *not* simultaneous." Even were it so, he alleges that *apparent* would be no adequate proof of *real* simultaneity; and for this reason, that states of consciousness which originally occurred in distinct succession, come to follow one another at last so rapidly by constant association as to seem inseparable, so that we unite a whole group of perceptions so instantaneously that they appear as one. He instances our inferring the unseen sides of a book, from the parts we see, and our inference of *solidity* from colour and extension. We cannot detect any interval between our perception of an object, and of its being distant from us, though the latter is evidently an inference from the former. Now the like may be the case in other instances of apparent simultaneity—from which, therefore, no decisive argument against cosmothetic scepticism, or absolute idealism can be drawn. Mr. Spencer repeats that we are still left to seek a *datum*—a test of the validity of the dicta of consciousness. But he first inquires into the bearing of Descartes' fundamental principle, "Cogito ergo sum," on this portion of his subject.

"Passing over all criticisms on the assumption that the proposition *I think* is more certain than the proposition *I am*—even granting that this last truth can become positively known only as a corollary from the first, there yet remains the fatal question—what gives validity to the therefore? Something more than the two states of consciousness, *I think* and *I am*, is involved; namely, the state of consciousness in which the relation of the one to the other is established. The absolute truth of the premises being admitted, it is clear that before absolute truth can be claimed for the conclusion, it must be proved to be absolutely true that the one involves the other. Surely this needs verification quite as much as the proposition *I am;* nay more, the cognition of the dependence of one thing upon another is more complex, and therefore more uncertain, than the cognition of either thing by itself."

From the above language, it would seem that the author understood Descartes' aphorism as designed to be a logical argument—a modern *enthymeme*—that is, a syllogism with one premiss (here the major) suppressed. So did Gassendi and other contemporaries understand him, as latterly Dr. Reid. Spinoza, in his "Principia," rightly apprehended his meaning, which indeed is evident enough, from the whole scope of Descartes' remarks on the subject; but he makes it decisive in his "Responsio ad Secundas Objectiones," where he says, in so many words, "I think, therefore, I am, or I exist, is not concluded by force of a syllogism, but as a thing self-evident." In fact, Descartes argues that he found he could doubt many other things, even the reality of external nature; but the very act of thought he felt to involve the irresistible conviction of the being of himself the thinker; and he adds, "I found that, in *je pense donc je suis*, there was nothing to induce me to believe it true excepting that I see clearly that, *to think, it is necessary to be.*" He concludes that "all those things which we conceive very clearly and distinctly are true"—an axiom which evidently lies widely open to the illusions of the imagination, to prejudice, and self-will; of which Descartes himself was in some degree aware, for he subjoins: "there is only some difficulty in well-marking what those things are which we conceive distinctly." Leibnitz was not satisfied with the Cartesian doctrine of "perfectly clear ideas," as ultimate and fundamental elements of belief: he calls for a test of this "clearness;" and he finds it in the "rules of logic," themselves resting on the principle of identity or contradiction.

But to return to Mr. Spencer's inquiry; he asks—"Is it not obvious that the first thing to be investigated is that mental act whereby we recognise the validity of our convictions?" We regard some convictions as less questionable than others, and some as unquestionable. We believe one thing rather than some other thing—why? "considering our whole knowledge to be made up of beliefs, the ground-problem is, to determine the nature of a true belief." He asks for a starting-point not in any one belief, but "in a *canon of belief*," in which lies the fact which underlies all other facts. He proceeds now to what he conceives to be the "desired result"—the "*Universal Postulate.*"

"In our search for this 'fundamental fact,'" says our author, "we meet the difficulty that several facts seem equally primordial—personal existence, the existence of ideas, of consciousness, of beliefs. Each seems to presuppose others; and yet each, in turn, seems first. One fact, however, being unavoidably taken for granted in every process of thought, must necessarily have priority of the others; namely, *belief.* Every logical act of the intellect is a predication, which is a belief; all connected thought being made up of beliefs, all the propositions it embodies must be less certain than the existence of beliefs, be they even the existence of consciousness, of ideas, of personality . . . Belief, then, is the fact which, to our intellects, is antecedent to and inclusive of all other facts. It is the form in which every fact must present itself to us, and therefore underlies every fact. It alone of all things cannot be denied without self-contradiction. The propositions—there is no consciousness, there are no ideas, there is no personal identity, may be absurd, but they are not immediately self-destructive. To say, however, there is no belief, is to utter a belief which denies itself; to distinguish between what is, and what is not, and at the same time to say that we do not so distinguish."

We suppose our author here to mean that a particular belief, distributively, may be less certain than the general fact of the *existence of beliefs.* We may be deceived in believing this or that, but it is impossible that in believing any proposition, event, or fact of consciousness, we can deny that there is such belief. So far so good. But we should demur to that part of the statement which maintains that the existence of consciousness and of ideas is "less certain than the existence of beliefs." Shall we say that consciousness is a form of belief, or that belief is a form of consciousness, or that they are both identical? It is worth while here to refer to Mr. Mansel's "Prolegomena Logica."*

* *Vide* "Psychological Journal," January, 1854.

He identifies every act of consciousness, in a certain sense, with judgment, there being always a conviction (belief) of the presence of the object of such act, either externally in space or internally in the mind—a conviction amounting virtually to the proposition, "This is here." Thus every operation of thought, even a *single* concept or idea, is a judgment, psychologically considered, though not logically, for a logical judgment requires two terms and the copula. On this principle, psychological judgments (convictions) must always precede logical ones, since we must apprehend (in the logical sense) the terms, before we can in any way compare them. On the same principle, all the spontaneous judgments of the mind are psychological; that is, all our actual intuitions, all the presentations of perception and imagination produce a realization of the presence of their objects without any logical process. Thus the Cartesian *ego sum* is a primitive psychological judgment; for self is so presented in consciousness, that to know what we mean by *ego* is to recognise the all-pervading conviction, sense, impression, belief—call it what we may—of our own existence: so that "*ego sum*" is rather an analytical than a synthetical judgment. To return to Mr. Spencer's statements: if we grant that consciousness is always a certain species of belief or conviction, a *quasi* judgment, not logical or deductive, but intuitive, spontaneous, and psychological (as above); still as consciousness is properly a modification of mind or self, and as from the very nature of the case we cannot but know, be convinced of, or *believe* the fact of this modification in any given instance—it is difficult to see why the beliefs of consciousness should (as Mr. Spencer thinks "is clear") be less certain than the fact of the "existence of beliefs." A man is conscious of suffering severe pain—what can be conceived more certain to him than this?

Our author having adopted the principle that belief is the ultimate fact in psychology, which we can never transcend, next asks—How do we class our beliefs? Why do we regard some of them as more trustworthy than others? and what is the peculiarity of those beliefs which we never question? He is unwilling to take for granted "consciousness, ideas, or personal identity;" and he proposes to assume only "existence, its co-relative non-existence, and a cognition of the difference—that is belief; the problem being to find a canon of belief, without assuming anything further." The discussion which follows reminds us of some modern German speculations; but this does not affect their merit one way or the other. "We may, by the union of the two terms existence and non-existence, obtain a third, which describes the nature of some of our beliefs as contrasted with others. Here is the only possible classification; beliefs of which existence alone can be predicated—that is, beliefs that *invariably exist;* and beliefs of which partly existence and partly non-existence can be predicated; that is, beliefs that *do not invariably exist.* All know that they have beliefs of which no effort can for a moment rid them; whilst they have also beliefs which are changed by evidence, or can be temporarily suppressed by the imagination." The conclusion is, that the *invariable existence of a belief is our final test of certainty.* We adopt it because we *must.* In saying that it is invariably existent, we say that there is no alternate belief.

A criticism follows, of the views of Dr. Whewell and Mr. Mill, respectively, on *necessary truth.* Our author seems to agree with Mr. Mill in his arguments in disproof of the "alleged *à priori* character of these necessary truths," and in his theory that "axioms are simply our earliest inductions from experience;" but he rejects Mr. Mill's criticism of Dr. Whewell's position, that truths are necessary, the negation of which is "inconceivable," though he denies with Mr. Mill that these truths, the negation of which is inconceivable, are *à priori.* In another part of the work, however, the author explains that he neither holds the experience hypothesis, nor the antagonist hypothesis of forms of thought, in their respective current acceptation. But for this discussion we have not space; nor for some acute criticisms on Sir W. Hamilton's objection to the test of inconceivability as the criterion of impossibility; namely, that "of two propositions, one of which must be true, this test proves both impossible. It proves that space cannot have a limit, because limited space is inconceivable, and yet that it must have a limit, because unlimited space is inconceivable; it proves, therefore, that space has a limit and has no limit, which is absurd." How absurd? asks our author. "Because," says Sir William, "it is impossible for the same thing to be and not to be." But how do we know that it is impossible, but because it is inconceivable? Our author adds, that space and time are not strictly conceivable things at all, in the abstract, in the sense that other things are—that the alleged inconceivableness of a minimum or a limit is not an inability to put one conception in the place of another, but an inability to form any conception—and that there is no true parallelism between the cases in which both alternatives are alike inconceivable, and all other cases, in which one alternative is conceivable and the other not. This mode of reasoning is at least worthy of consideration.

The author now rehearses, that the existence of beliefs is the fundamental fact—that beliefs which invariably exist are those which, both rationally and of necessity, we must adopt; and that the *inconceivability of its negation is the test by which we*

*ascertain whether a given belief invariably exists or not.* When such a belief exists, we have no "alternate belief," which we may know by trying to put some other belief in its place; that is, by trying to conceive the negation of it. When nothing else is conceivable, it is unquestionably true. "Instinctively we recognise the truth above demonstrated, that *its invariable existence is the ultimate authority for any belief.*" This last remark, we suppose, is itself a belief—or rather two beliefs—one being that invariableness is our authority for belief; and the other belief being that we *instinctively* recognise this test of invariableness. The term "intellectual instinct" is not unknown to psychological usage, and it signifies, we suppose, the same as intuition, in the English sense (a wider sense than that of the German *anschauung*). It points to a perception of truth, of which no account can be given. We might be said to believe in our being, and in the facts of consciousness, on this principle, perhaps as correctly as on any other. At all events, is not the use of the term "instinctively," used by Mr. Spencer in reference to our "recognising truth," a virtual giving up of the test, in the innermost adytum of the citadel? Is it not a falling back at once upon a blind impulse, or necessity of our mental constitution? Does it not, at all events, in the last resort, identify this test of "invariability" with an irresistible constitutional tendency to believe some things—we know not why—excepting that we cannot help it. The least inference that we can draw from the above is, that it is very difficult to adhere consistently to *terms*, in the attempt to explore the shadowy region of our ultimate mental principles.

By whatever name we may call this irresistible necessity we feel of believing *some things*—whether it be described as a kind of "instinct," as natural impression, primitive belief, or by any other term, whether it be in any case simply spontaneous and psychological in its form, or in any other case more logical and synthetic; whether, as according to Reid and Kant, such elemental convictions are *à priori*, or, as Mr. Mill insists, with the assent of our author, "axioms are simply our earliest inductions from experience" (to which statement as general we should demur), we fully agree with him that such beliefs must be their own sureties—that an indestructible belief can have no other warrant than its own indestructibility. Mr. Mill has shown, in his "System of Logic,"* that there are remarkable instances in the history of science, in which things were rejected as impossible, because inconceivable, which everybody now knows to be true. The "experimental proof of the indestructibility of a belief, found in the inconceivableness of its negation,"

* pp. 265, 266.

as proposed by Mr. Spencer, requires, therefore, a very careful application. Our author's main reply to Mr. Mill is, that the very facts which he adduces show that "men have mistaken for inconceivable things, some things which were not inconceivable." Examples of beliefs invariably and indestructibly existing are adduced; indeed, such are all the truths of immediate consciousness. They have no other warrant. Such is the truth "I am"—the fact of any particular sensation at a given time. As long as personal existence lasts, the denial that we exist is inconceivable. So, while feeling a sensation of coldness, we cannot possibly conceive that we are actually not feeling it.

The author next states his views respecting what he conceives to be the "real distinction between those universal truths which Dr. Whewell has supposed to stand alone in the inconceivableness of their negations, and those particular truths which we find to have the same guarantee." "How," he asks, "does the belief 'this is sunlight' differ from this—'the whole is greater than its part?'" He replies, "Simply in this—that in the one instance the antecedents of the conviction are present only on special occasions; in the other instance, on all occasions. In either case only one belief is conceivable. In the one case, a single object serves for antecedent; in the other, any object, real or imagined." In the same way, every *axiom* and every demonstration may be shown, either immediately or finally, to be warranted only by the same invariable belief. But we have beliefs which, though strong, do not invariably exist. The belief that the sun will rise to-morrow, is very commonly regarded as a constant one; but we may suppose the prevention of this event possible in many ways. Mr. Spencer says, that while we are imagining these possibilities, "the belief that the sun will appear is non-existent; though speedily reproduced, it is nevertheless temporarily annihilated." To suppose the belief to remain, even while we are conceiving the event to be otherwise, "is an illusion consequent on our habit of using words without fully realizing their meanings, and so mistaking verbal propositions for real ones." "We cannot conceive the event of the sun's rising to-morrow otherwise than it is; that is, conceive of its non-occurrence, without abolishing the representation of its occurrence—abolishing the belief." We speak commonly of a belief as something separate from the conception to which it relates, but the belief is nothing more than the "persistence of the conception." We have a weak belief when we can easily change a state of consciousness which has arisen after given antecedents; we have a strong belief when we can only change such state of consciousness with difficulty; we have a belief of the highest order when we are utterly unable to change it. In each case the belief merely expresses the persistence of a certain state of consciousness. "The belief being the persistence, the persistence cannot be destroyed, even temporarily, without the belief becoming non-existent for a corresponding period." "If the persistence of the state of consciousness can be broken, the belief is thereby proved to be not invariably existent." It is worth while for the reader, who has the opportunity, to compare this whole discussion on Belief with a chapter on the same subject by Mr. Mill the elder.* There are many coincidences of view, though the scope of the respective writers is not exactly the same. Mr. James Mill's aim is, throughout, to prove the identity of Belief and Association. We will now quote Mr. Spencer's statement respecting the result of the above inquiries, which conduct him to the principle already enunciated, and which he terms the UNIVERSAL POSTULATE :—

"Returning to the purely abstract view of the matter, we see—first, that belief is fundamental, and that the invariable existence of a belief is our highest warrant for it; second, that we can ascertain the invariable existence of a belief only as we ascertain the invariable existence of anything else, by observing whether under any circumstances it is absent from the place in which it occurs; third, that the effort to conceive the negation of a belief, is the looking in the place in which it occurs—namely, after its antecedents—and observing whether there are any occasions on which it is absent, or can be made absent; and, fourth, that when we fail to find such occasions—when we perceive that the negation of the belief is inconceivable, we have all possible warrant for asserting the invariability of its existence; and in asserting this, we express alike our logical justification of it, and the inexorable necessity we are under of holding it. Mean what we may by the word truth, we have no choice but to hold that *a belief which is proved by the inconceivableness of its negation to invariably exist, is true.* We have seen that this is the assumption on which every conclusion whatever *ultimately* rests. We have no other guarantee for the reality of consciousness, of sensations, of personal existence; we have no other guarantee for any axiom; we have no other guarantee for any step in a demonstration. Hence, as being taken for granted in every act of the understanding, it must be regarded as the *Universal Postulate.*"

Having thus laid down his Universal Postulate for Truth and Belief, Mr. Spencer comments on Mr. Mill's argument above referred to—namely, that on past occasions this postulate has proved insufficient, and may prove so again. Things once thought inconceivable are now universally and fully believed. The great philosophers referred to by Mr. Mill, who were incredulous of the existence of antipodes, because they could not, in opposition to old associations, conceive of the force of gravity acting "upwards instead of downwards," were dealing, replies

* "Analysis of the Phenomena of the Human Mind," by James Mill, Esq. 1829. Vol. i. chap. 11.

Mr. Spencer, with many states of consciousness, and with the connexions between them. The concepts, earth, man, distance, position, force, and various relations of these to each other, entered into their proposition.

"We must distinguish," he remarks, "between those appeals to the Universal Postulate in which the act of thought is *decomposable*, and those in which it is *undecomposable*. In proportion as the number of concepts which a proposition involves is great, and the mental transitions from concept to concept are numerous, the fallibility of the test will increase, and will do this, *because the formation of the belief is divided into many steps, each of which involves the postulate*."

The theory here, as further stated by the author, is, that, as we are liable to mental *lapsus*, we shall occasionally think we have the warrant of the postulate, when we have it not; and our liability to being thus deceived will vary as the number of times we have claimed its warrant. Our author says that we "instinctively recognise this fact in our ordinary modes of proof"—(again, it is observable, he makes instinctive or spontaneous tendencies the same things as beliefs). An example of the alleged fact is introduced, which does not appear to us a very happy one. "We hold it more certain," says Mr. Spencer, "that 2 and 2 make 4, than that 5 + 6 + 7 + 9 + 8 make 35;" and he seems to mean what he says. But is this true? Properly understood, we think not. We are reminded, by this example, of Kant's views of similar arithmetical propositions, which he regards, and justly, as equally "synthetical," whether the subject consist of two terms, or any number whatever. Surely, we may at all events assert that nobody who understands addition would admit that two numbers correctly added more *certainly* amount to their sum, than any series of numbers, however long, correctly added, amount to *theirs*. We can easily understand that it is easier to add two numbers together than to add five, and that there is just four times as much risk of error in the operation: and if this is all that is meant, we must admit Mr. Spencer's statement. But nobody, if the condition of the *plus* sign be attended to—in other words, if the addition be actually (*i.e.* correctly) made—would hold it more certain that 4 was the result of one addition, than that 35 was the result of the other. In taking his crucial example from the exact sciences, in which every succeeding step in the demonstration is equally certain, if that step be really taken, Mr. Spencer, as appears to us, has hardly done justice to his theory of the Postulate. The case of the "antipodes" is widely different. We might as well say that, on the supposition that a man shall actually walk from the beginning to the end of a given straight line, it is more certain that he will reach any other given point in it than the extremity! Our author, however, draws to a conclusion his preceding remarks, as follows:—

"Not only as judged instinctively, but as judged by a fundamental logic, that must be the most certain conclusion, which involves the postulate the fewest times. We find that, under any circumstances, this must hold good. Here, therefore, we have a method of ascertaining the respective values of all cognitions."

This last remark seems to call for some comment. What distinction does the author intend to draw between "instinctive judgment" and "fundamental logic?" We suppose "instinctive judgment" to mean the same thing as "invariable, indestructible belief." On what then, different from this, do the fundamental laws of logic rest? Are not these laws invariable, indestructible beliefs? What else are the axioms, that "if two terms agree with one and the same third, they agree with each other;" and "if one term agrees and another disagrees with one and the same third, these two disagree with each other?" If it be said that "instinctive judgment," or common sense, perhaps, leads to any conviction, how else do the laws of logic lead to it? And with regard to the touchstone itself, of the "respective values of all cognitions"—namely, the fewness of the times the Postulate is involved—it would, if we understand Mr. Spencer's views aright, be fatal to the certainty of the exact sciences, in which conclusions are often reached by a considerable chain of propositions, all following rigorously from each other.

Mr. Spencer proposes, in his third chapter, to exhibit the "chief corollaries" involved in his Universal Postulate, by putting to this test some of the principal metaphysical theories which have prevailed. We have some criticisms on Berkeley:—

"Self-consciousness cannot be immediate knowledge," says our author. "We can only be conscious of what we were a moment ago. Nothing can be immediate knowledge into which self-consciousness enters as one concept. Therefore, the knowledge of having sensations cannot be immediate knowledge. Were the consciousness of sensations the same thing as the consciousness of receiving sensations, Berkeley's first step would be unassailable:"

that is, the principle that what we call external objects are our own sensations, of which we have immediate knowledge, and in feeling which we cannot be mistaken, would be unassailable. Our author thinks that Berkeley "confounds the *having a sensation* with the *knowledge* of having it." We do not see that this distinction, or the author's remarks, above quoted, on self-consciousness—granting their validity—can affect the case either way. The dispute was about the *cause* of our sensations. Berkeley never doubted that they had a cause without us: he only referred them immediately to the causation of the First Cause—an "Eternal Spirit," denying the existence and even possibility of any secondary or intermediate causation, such as we assign to what we call matter, by a natural unvarying instinct, a constant impression or belief existing from infancy to death—a conviction inseparable, even by reasoning, from our animal life—maintained in action every moment by the perpetual sense we have of resistance—a resistance which often overcomes our most strenuous acts of will, and consequent muscular exertion.

We have some acute and elaborate criticisms in connexion with Hume's Scepticism:—

"Which is the more certain," asks our author, "the existence of objects, or the existence of impressions and ideas? How is this question to be decided? The reply is: The relative validity of our beliefs in subjective and objective things is tested by the number of times the Universal Postulate is assumed in arriving at each belief respectively."

We have already intimated the difficulty of abiding by this general principle, and we cannot here pursue this point. Mr. Spencer, however, ingeniously illustrates his doctrine by the example of our looking at an object—a "book," for instance. We do not think about any sensation, we attend only to the book. We know nothing of any image of it;—we are "conscious of the book," not of an impression of the book—of an objective thing, and not of a subjective thing. The sole content of our consciousness is the book, considered as an external reality. We feel that our recognition of the book is an indecomposable act. We cannot, while contemplating the book, believe that it is non-existent: while we continue looking, the belief in the existence of the book possesses the highest validity possible. "It has the direct guarantee of the Universal Postulate, and it assumes this Postulate only once." No doubt, the main impression is here the psychological, not logical one—equivalent to the "book is here," as Mr. Mansel would express it.*

Certainly, our own being does not seem here to be consciously postulated; and this is the reason why we think a distinction, not always made, should be observed between general consciousness and self or reflected consciousness. Mr. Spencer here objects to Sir W. Hamilton's view, that in perception we are conscious of subject and object "in the same indivisible moment of intuition"—not that he objects to extending the term consciousness to what is not actually within our own minds, but because in many cases our minds are so absorbed in the object that there is no accompanying consciousness, at the time, of subjective

* "Prolegomena Logica."

existence. He appeals, not without reason, to ordinary language in support of this (as we think) undeniable fact:—We are "absorbed" in thought, "lost" in wonder; a man has "forgotten" himself. Men sometimes lose their lives by their attention being wholly occupied by something else when danger is present. Again, the infant's earliest perceptions are unaccompanied by any notions of self. Our author concludes that the cognition of an object as an external reality is an undecomposable mental act, involving the Universal Postulate once only, and therefore it has the highest certainty.

Hypothetical Realism, or Cosmothetic Idealism—(*i.e.*, the doctrine that the external world is not directly cognized in consciousness, but by some form of representationism)—is next tested by the postulate. A man looks at a fire. If it be said, he can only know his own impression of the fire, what follows? "He postulates the fire; he postulates himself; and he postulates the relation between these. Instead of an immediate undecomposable cognition of the fire, the alleged representative cognition seems decomposable at once into three things, and cannot be conceived without them. In the one case,* he cannot use the Postulate more than once; in the other, he cannot avoid using it three times." The conclusion is, that we have, thus, a proof that Natural Realism is more certain than any form of Representationism. But, independently altogether of the claims of the various theories of external perception, we fear that this test of the Postulate would sometimes prove what is contrary to consciousness. For there are cases, surely, in which our attention is especially directed to the relation which there is between the *ego* and the *non-ego*. We may, for instance, speculate or experiment on the properties of matter with immediate *reference* to the sensations it produces in us. Shall we, then, say that because three things are implied—namely, the object, the *ego*, and a relation between them—we are not as certain of the relation as we are of the object? Suppose the man to try how near he can go to the fire without changing the agreeable warmth he feels, into the pain of incipient scorching—is he not as certain of the relation between his sentient self and the fire, as he can be of the fire's existence in any theory of perception? Surely he is, and justly. Yet the belief in this relation—which is so firm, involves the two other elements. Our author, in further applying his test to Absolute Idealism, distinctly holds that if a proposition or theory be based on three beliefs, each confessedly "indisputable," it would be less certain than though it stood on one such belief. This we cannot agree to. It would prove that

* The case of "Natural Realism," "Presentationism," or "Intuitionism." See Sir W. Hamilton's "Reid," p. 816, *seq.*

the axioms of Euclid were more really certain than the results of the demonstrations which assume them; but who would venture to say this?

The author subjects the Kantian hypothesis with regard to the entire subjectivity of space and time to the Postulate, and shows that it is opposed to our invariable natural belief, and is wholly inconceivable in connexion with the real existence of things (*Ding an sich*), which Kant inconsistently admitted. Many of the speculations of Fichte, Schelling, and Hegel are obviously incapable of maintaining their ground against the same test. They are at variance with invariable, indestructible belief.

We regret that we cannot proceed with the remainder of this elaborate work. We have ventured to differ from the author respecting the *application* of his fundamental principle—the Universal Postulate; but we should do him injustice if we did not state that our analysis of this principle necessarily fails to convey to the reader an adequate idea of the work as a whole. The bulk of the volume consists of discussions, which rarely contain any explicit reference to the Postulate; after the explanation of which, follows a profound treatise on Compound Quantitative Reasoning, as illustrated by the laws of mechanics, the doctrine of ratios, and by geometrical and algebraical analysis. Another Dissertation follows on Qualitative Reasoning, perfect and imperfect; that is, reasoning into which the idea of co-extension does not enter as a necessary element, and by which co-existence and non-coexistence are determined. Next is a chapter on Reasoning in General, in which Mr. Spencer denies that the Aristotelian dictum *de omni et nullo*, or Mr. John Mill's axiom that "whatever possesses any mark possesses that which it is a mark of," or any other axiom that can possibly be framed, can express the ratiocinative or inferential act, which, he says, is a "single and almost unconscious intuition;—a description of it to which we do not assent;" for if so, we do not see how we could be said to "infer" at all, any more than we *infer* that we have a sensation of pain when we can at once reduce our sense of it to a single intuition.

Dissertations follow on Perception, the Relations of Things, and Consciousness; and these are succeeded by a most elaborate analytical inquiry into the physiological and psychical Connexion of Mind and Life; all which are well worthy of the student's attention; though he may find in some of them a greater dependence of mind on bodily organization than he would expect, especially considering that phrenology as a system is here rejected. The work closes with a chapter on Intelligence, Reflex Action, Instinct, Memory, Reason, the Feelings

(emotions), and the Will. The whole volume, to the student who has been accustomed to Reid and Stewart, and to the Eclectic and Kantian doctrine of *à priori* and synthetic truth, will probably appear of a too experimental and "sensational" cast. Our author thinks that all mental phenomena are only "incidents of the correspondence between the organism and its environment;" and he maintains that there is "really no line of demarcation between reason and passion." He holds with Mr. Mill, that axioms, or so-called necessary truths, are simply our earliest inductions from experience. On the Will and its Freedom, our author's opinions will appear specially objectionable. He regards the current opinions on these subjects as illusory; and many will think that his theory tends to a species of fatalism.

"All actions," he remarks, "must be determined by those psychical connexions which experience has generated, either in the life of the individual, or in that general antecedent life whose accumulated results are organized in his constitution. . . . . The changes which he is said to will are wholly determined by the infinitude of previous experiences, so far at least as they are not produced by immediate impressions on the senses. . . . A body in space, subject to the attraction of a single other body, will move in a direction that can be accurately predicted. If it is surrounded by bodies of all sizes, in all directions, at all distances, its motion will be apparently independent of the influence of any of them; it will move in some indefinable varying line that appears to be self-determined; it will seem to be free. And, in the same way, just in proportion as the cohesions of each psychical state to others becomes greater in number and various in degree, the psychical changes will become incalculable, and apparently subject to no law. . . . . If psychical changes do conform to law, there cannot be any such thing as free-will."

This denial of free-will is contrary, we think, to the "Universal Postulate;" for surely we are irresistibly and invariably conscious that we are free. But we must forbear further comment, and we only add that, though we have met with many things in the work from which we have been obliged to withhold our assent, we can nevertheless commend it to the reader's attention as a work of high talent, deep research, and great analytical power. It is an important contribution to speculative philosophy, and well deserves a diligent and careful study.

